# Editorial: Mechanisms and novel treatments of pigmentary disorders and skin regeneration

**DOI:** 10.3389/fmed.2022.1016741

**Published:** 2022-09-14

**Authors:** Xing-Hua Gao

**Affiliations:** Department of Dermatology, The First Hospital of China Medical University, Shenyang, China

**Keywords:** pigmentary abnormality, skin, regeneration, hypopigmentation, hyperpigmentation

Pigmentary disorders, whether hyperpigmentary or hypopigmentary, are quite common dermatologic conditions (1). Depending on the extent and location of involvement, patients with pigmentary disorders may experience poor quality of life and psychosocial status owing to the disfiguring effect of these conditions and, occasionally, their systemic involvement.

The present issue collected two studies on rare genetic defects in pigmentary disorders from Chinese descendants. *KIT* is a proto-oncogene that is involved in the proliferation, survival, and regulation of melanocytes, among a couple of other cells. Yang et al. presented a case study of a three-generation Chinese family with progressive hyperpigmentation and generalized lentigines, inherited in an autosomal dominant pattern. A missense mutation of c. 2485G > C in the KIT gene was identified. Geno-pheno-correlation analysis was suggestive of cutaneous pigmentary changes without systemic involvement.

GS type 3 (GS3) is a very rare autosomal recessive disorder caused by mutations in the melanophilin (MLPH) gene, characterized by pigmentary dilution of skin and hair with no immunological or neurological manifestation. The MYO5A-MLPH-RAB27A ternary protein complex is required for anchoring mature melanosomes in the peripheral actin filaments of melanocytes for subsequent transfer to adjacent keratinocytes. Huang et al. identified a novel homozygous missense mutation (c.73G > C; p.D25H), residing in the conserved Slp homology domain of MLPH, in a Chinese GS3 patient who presented with hypopigmentation of the hair, eyebrows, and eyelashes.

Treatment of pigmentary disorders is challenging. Post-inflammatory hyperpigmentation (PIH) is a common acquired pigmentary disorder following skin inflammation and exaggerated by ultraviolet B (UVB) light. Zhao et al. demonstrated that a steroidal alkaloid glycoside, solamargine, exhibited an anti-inflammatory effect through the p38 MAPK/Nrf2/HO-1 signaling pathway and an inhibitory effect on UVB-induced melanin synthesis. It is tempting to test the *in vivo* effect of solamargine in PIH in future.

Melasma is a common acquired hyperpigmentation disorder characterized by hyperpigmented patchy skin in sun-exposed areas. Topical botanical products containing active ingredients have been increasingly applied as therapies for melasma. Wang et al. conducted a systemic review and meta-analysis of randomized controlled trials on agents with either herb-derived molecule, extracts of a single herb, or extracts of compound herbs. Of the 12 included studies, all showed superior effects in reducing the severity of melasma when compared with placebo, and better safety profiles compared to active controls. The authors assumed that the mechanisms of action are (1) direct or indirect inhibition of tyrosinase to suppress melanogenesis; (2) antioxidation to scavenge free radicals; (3) regulation of inflammatory mediators to inhibit inflammation; or (4) synergistic action of the above. Although these results are appealing, issues surrounding the characterization of specific molecules remain. Well-designed clinical trials are still needed, as well as observations on the long-term efficacies of these compounds.

Vitiligo is an autoimmune depigmentation disorder caused by the destruction of melanocytes, which is multifactorial in origin. Chang et al. conducted a combined therapy consisting of camouflage and psychotherapy on patients with vitiligo and demonstrated a significant improvement in their quality of life. Serum levels of neuropeptide-Y and melanin-concentrating hormone significantly decreased, and levels of adrenocorticotropic hormone increased, in treated patients but not in the control group. The serum levels of interferon-γ, CXC chemokine ligand 10, and interleukin-1β decreased in both the active and stable stages of the intervention group and only in the active stages of the control group. Non-randomized design and a lack of reporting on clinical improvement are the major limitations of this study.

Skin regeneration therapies aimed at the prevention and reversal of skin aging are greatly hoped for by the public. Mesenchymal stem cells (MSCs) are multipotent stem cells deriving from an array of sources such as bone marrow, adipose tissue, or umbilical cord blood. MSCs, as well as their derivatives, may promote cell proliferation and neovascularization in skin regeneration, decrease inflammation in injured skin, produce collagen and elastic fibers, inhibit metalloproteinase activation, and avoid UV-induced senescence (2). Wang et al. reviewed the mechanisms of action of extracellular vesicles secreted by adipose-derived MSCs on skin regeneration, skin barrier repair, and hair growth, specifically on their roles in inflammation, angiogenesis, cell proliferation, extracellular matrix remodeling, autophagy, and oxidative stress. Liang et al. conducted a randomized, controlled split-face study on volunteers with facial skin aging and demonstrated that the combination therapy of microneedling and conditioned media of human umbilical cord-derived MSCs was beneficial for skin rejuvenation. A major limitation of the study is that specific active molecules as well as potential hazardous molecules were not defined in the culture media.

A wound is defined as an injury or disorder in the normal skin structure, inducing discontinuous body tissue. Wound healing is an overlapping processes, and is divided into the stages of inflammation, blood clotting, cellular proliferation, and extracellular matrix remodeling (3). Xiong et al. constructed a rat scalp-expansion model and found that topical application of metformin enhanced the proliferative activity of skin-derived stem cells, as well as the regenerative capacity of mechanically stretched skin, as shown by increased epidermal thickness, collagen volume, and angiogenesis. Mao et al. reviewed the pivotal role of follicular stem cells in epithelial-mesenchymal interaction in the context of hair follicle regeneration and skin wound healing, emphasizing their control in the exchange of soluble molecules, modulation of key pathways, and signal transduction via the extracellular matrix.

To sum up, the current issue covers nine papers comprising original studies and reviews on pigmentary disorders and skin regeneration. These studies added new data on the genetics of a couple of rare pigmentary diseases and on the role of stem cells and their derivatives in skin regeneration. Several of these pioneering trials deserve attention and follow-up in the future.

## Author contributions

The author confirms being the sole contributor of this work and has approved it for publication.

## Conflict of interest

The author declares that the research was conducted in the absence of any commercial or financial relationships that could be construed as a potential conflict of interest.

## Publisher's note

All claims expressed in this article are solely those of the authors and do not necessarily represent those of their affiliated organizations, or those of the publisher, the editors and the reviewers. Any product that may be evaluated in this article, or claim that may be made by its manufacturer, is not guaranteed or endorsed by the publisher.
